# Optimizing the Distillation of Greek Oregano—Do Process Parameters Affect Bioactive Aroma Constituents and In Vitro Antioxidant Activity?

**DOI:** 10.3390/molecules28030971

**Published:** 2023-01-18

**Authors:** Alexandros Nakas, Georgia Giannarelli, Ioannis Fotopoulos, Eirini Chainoglou, Aikaterini Peperidou, Konstantinos N. Kontogiannopoulos, Artemis Tsiaprazi-Stamou, Vasilios Varsamis, Helen Gika, Dimitra Hadjipavlou-Litina, Andreana N. Assimopoulou

**Affiliations:** 1 Laboratory of Organic Chemistry, School of Chemical Engineering, Aristotle University of Thessaloniki, 54124 Thessaloniki, Greece; 2 Natural Products Research Centre of Excellence, Center for Interdisciplinary Research and Innovation of Aristotle University of Thessaloniki, 57001 Thessaloniki, Greece; 3 Department of Pharmaceutical Chemistry, Faculty of Health Sciences, School of Pharmacy, Aristotle University of Thessaloniki, 54124 Thessaloniki, Greece; 4 Vessel Essential Oils, Neo Rysio, 57001 Thessaloniki, Greece; 5 School of Medicine, Aristotle University of Thessaloniki, 54124 Thessaloniki, Greece

**Keywords:** oregano, essential oils, design of experiments, headspace GC-MS, antioxidant activity

## Abstract

The aim of the present work was to optimize the conditions of the distillation process at a pilot scale to maximize the yield of specific bioactive compounds of the essential oil of oregano cultivated in Greece, and subsequently to study the in vitro antioxidant activity of these oils. Steam distillation was conducted at an industrial distillery and a Face-Centered Composite (FCC) experimental design was applied by utilizing three distillation factors: time, steam pressure and temperature. Essential oil composition was determined by static headspace gas chromatography–mass spectrometry (HS–GC/MS). To obtain a comprehensive profile of the essential oils, instrumental parameters were optimized, including sample preparation, incubation conditions, sampling process, injection parameters, column thermal gradient and MS conditions. With the applied GC-MS method, more than 20 volatile compounds were identified in the headspace of the oregano essential oils and their relative percentages were recorded. Carvacrol was the most prominent constituent under all distillation conditions applied. Data processing revealed time as the main factor which most affected the yield. The Desired Space (DSc) was determined by conducting a three-dimensional response surface analysis of the independent and dependent variables, choosing yields of thymol and carvacrol as optimization criteria. The in vitro antioxidant activity of the essential oils of all samples was measured in terms of the interaction with the stable free radical 2,2-diphenyl-1-picrylhydrazyl (DPPH) after 20 and 60 min. The most prominent essential oils at different distillation conditions were also tested as inhibitors of lipid peroxidation. Higher % values of carvacrol and thymol were correlated to higher antioxidant activity. Evaluating the impact of the distillation conditions on the in vitro results, it seems that lower pressure, less time and higher temperature are crucial for enhanced antioxidant activities.

## 1. Introduction

The common name oregano is ascribed to more than 60 plant species of the Lamiaceae family, along with members of other families, such as Verbenaceae. Among the different Lamiaceae species, *Origanum vulgare* L. (section *Origanum*) is the most prominent oregano representative in the food and pharmaceutical industry [1,2].

Plants belonging to the *Origanum* genus are known for their medicinal uses, as well as being culinary plants since ancient times. *Origanum vulgare* L. subsp. *hirtum* (Link) Ietswaart is an aromatic, rich in essential oil [3], perennial sub-shrub, widely distributed all over Eurasia and North Africa [4]. It is called Greek oregano, and it is endemic across the Mediterranean region, cultivated across most of the world and is regarded as the most valuable oregano [5]. Another subspecies important from the economic point of view is *Origanum vulgare* L. subsp. *vulgare* (common oregano) [6,7].

Greek oregano is rich in essential oil (more than 1.1%, based on previous studies), while common oregano contains a lesser amount (not exceeding 0.8% of dry weight) [8]. Subspecies exhibit a few chemotypes defined on the basis of the dominant compound in the essential oil. Greek oregano accumulates mainly phenolic monoterpenes (thymol and carvacrol), followed by their precursors (p-cymene and γ-terpinene). A substantial quantity of carvacrol, which is responsible for the characteristic “oregano” flavor, is very often detected in the essential oil of *Origanum vulgare* L. subsp. *hirtum* [1,2,6,9]. Both subspecies accumulate also significant amounts of phenolic compounds such as flavonoids and phenolic acids (rosmarinic, caffeic, vanillic, o-coumaric and protocatechuic acids) [10,11,12]. In relation to this wide variety of bioactive compounds, both *Origanum* subspecies indicate various pharmacological activities, especially antimicrobial, choleretic and antioxidant. In addition, they are widely used as culinary herbs, food preservatives and as flavoring and cosmetic ingredients [13,14]. Plant-derived foods and their essential oils are one of the main groups of foods that possess a potential antioxidant effect. The antioxidant activity of oregano’s essential oil (although the plant material was not botanically characterized) was associated with the predominance of carvacrol and thymol [15], the major components of the essential oil of *Origanum vulgare* L. subsp. *hirtum* [8,16,17]. This oregano’s essential oil has antioxidant properties effective in retarding the process of lipid peroxidation in fatty foods, and in scavenging free radicals [18]. These results indicated that the antioxidant effect may be related to the presence of phenols in the essential oil and that, besides thymol and eugenol [19], other phenols present in essential oils may also have an antioxidant effect.

*Origanum vulgare* subspecies have been shown to present high antioxidant capacity in a number of antioxidant assays, including interaction with the stable free radical 2,2-diphenyl-1-picrylhydrazyl radical (DPPH), the scavenging of cationic radical 2,2′-azino-bis(3-ethylbenzothiazoline-6-sulfonic acid) (ABTS) [20], ferric reducing antioxidant power (FRAP) and oxygen radical absorbance capacity (ORAC) [21,22], which is attributed to the high content of phenolics such as rosmarinic acid, eriodictol, naringenin and epicatechin [23]. Koldaş et al. associated the antioxidant capacity of *Origanum vulgare* species principally with the presence of rosmarinic, chicoric and caffeic acid [24].

It is well known that the extraction method and the process parameters highly influence the quality of the essential oils, as well as their bioactivity [25,26]. The method and conditions utilized must protect the beneficial components of essential oils from being decomposed or oxidized. Essential oils are typically produced commercially by steam distillation or hydrodistillation. Environmentally friendly methods such as supercritical fluid extraction (SFE) and microwave extraction have been also developed [27]. The antioxidant and antimicrobial activities of essential oils obtained from *Origanum vulgare* subsp. *hirtum* were determined by using solvent-free microwave extraction (SFME), supercritical fluid extraction and conventional hydrodistillation (CH) methods. It seems that the scavenging effects of essential oils obtained from oregano by using SFME and CH on the ABTS were similar. However, essential oil extracted by CH showed higher (2.69 mmol/mL of oil) Trolox equivalent antioxidant capacity (TEAC) than oregano oils obtained by SFME [27].

Industrial distillation of aromatic plants is usually performed at process parameter values defined mostly empirically and with the only criterion being the yield of the essential oil. The aim of the present work was to optimize the conditions of the distillation process to simultaneously maximize the total yield and the yield of specific bioactive compounds of the essential oil of oregano (*Origanum vulgare* subsp. *hirtum*) cultivated in Greece, and to subsequently study the in vitro antioxidant activities of these oils. For this purpose, a Face-Centered Composite (FCC) experimental design was applied to optimize the three main distillation factors (i.e., duration of distillation, steam pressure and temperature). Essential oil composition was determined by headspace GC-MS. The in vitro antioxidant activity was measured in terms of the interaction with the stable free radical DPPH after 20 and 60 min, while samples with encouraging results were further tested as inhibitors of lipid peroxidation.

## 2. Results and Discussion

### 2.1. Distillation Yields

For every distillation that was performed, 6 kg of dried plant material was used. At the end of each distillation process, the acquired essential oil was weighed and the respective yield (% *w*/*w*) was recorded (Table 1). The range of yields observed was rather wide: the minimum value was 0.3%, while the maximum yield reached 2.7%. The 32 independent distillation processes of the first stage averaged a yield of 1.44% with a standard deviation of 0.78%. Furthermore, by looking across the independent samples obtained through the same set of distillation parameter values (e.g., Std 1 and 2, Std 29 through 32, etc.), it seems that the reproducibility was almost perfect. It is also apparent that short-run distillations corresponded to low yields (0.3–0.7% *w*/*w*), while prolonged processes corresponded to much higher yields (2.1–2.7% *w*/*w*), with duration times in-between them yielding intermediate values (1.2–1.6% *w*/*w*).

### 2.2. Headspace GC-MS Analysis

**Figure 1:**
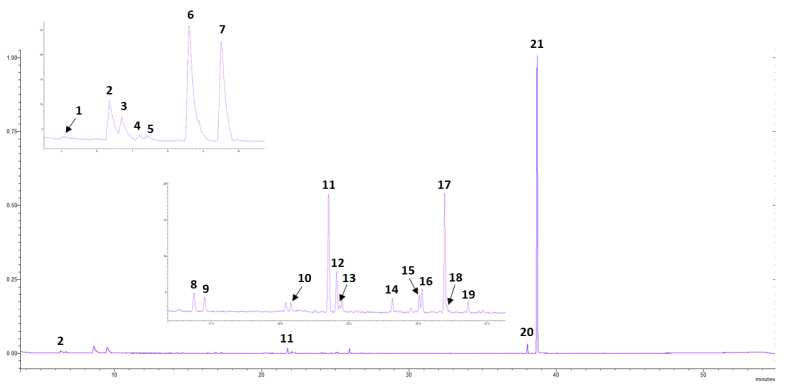
Typical GC-MS total ion chromatogram (TIC) of oregano’s headspace part of the essential oil. The numbers in the insets represent the detected components: (1) β-pinene, (2) β-myrcene, (3) α-terpinene, (4) limonene, (5) β-phellandrene, (6) γ-terpinene, (7) p-cymene, (8) 1-octen-3-ol, (9) 4-thujanol, (10) linalool, (11) β-caryophyllene, (12) terpinen-4-ol, (13) dihydrocarvone, (14) α-caryophyllene, (15) α-terpineol, (16) endo-borneol, (17) β-bisabolene, (18) carvone, (19) δ-cadinene, (20) thymol, (21) carvacrol.

In total, 32 distillations were carried out at the optimization stage, and 3 more after data processing, in order to validate the proposed model and the optimum conditions. With the aforementioned GC-MS method, we were able to identify and quantify 21 components of the oregano’s essential oil. An indicative total ion chromatogram is shown in Figure 1, where the 21 compounds have been tagged.

The 32 chromatograms obtained had a relative uniformity, and no large differences could be easily observed in terms of the peaks detected, due to identical plant material being used for the distillation process. However, despite the optical similarity among the chromatograms, there was still significant variation in some compounds, which was demonstrated after peak integration (Table 2).

The majority of the identified compounds were present in all samples. The most abundant component of the essential oils obtained, was, by far, the phenolic monoterpenoid carvacrol. Other compounds in relatively high abundance were γ-terpinene and p-cymene, followed in most cases by thymol, β-myrcene, α-terpinene, β-caryophyllene and β-bisabolene. The 13 remaining peaks were identified as follows: β-pinene, limonene, β-phellandrene, 1-octen-3-ol, 4-thujanol, linalool, terpinen-4-ol, dihydrocarvone, α-caryophyllene, α-terpineol, endo-borneol, carvone and δ-cadinene. Each one of them typically did not exceed 0.5% of the total ion chromatogram (TIC). All identified compounds, as well as their relative abundance in the headspace fraction οf the 32 oregano essential oils, are shown in Table 2.

As already mentioned, under all process parameters applied, the most prominent constituent—by a considerable margin—was the phenolic monoterpenoid carvacrol. This finding is in agreement with the available literature, where it is also stated that the predominant participation of carvacrol in the essential oil is responsible for the characteristic “oregano” flavor [1,2,6,8,9,16]. In a recent paper, after reviewing all published data for *Origanum vulgare* subsp. *hirtum* grown wild in Greece, Mertzanidis et al. concluded that this taxon is always rich in the isomeric phenolic monoterpenoids carvacrol and thymol, which are accumulatively responsible for 55–94% of the total oil content (when analyzed with direct GC-MS injection) [8]. In these plants, the antioxidant activity of the essential oil is associated with the predominance of carvacrol and thymol [15], which seem to exhibit comparable antioxidant activity [16].

In the present study, the relative abundance of carvacrol in the volatile fraction of the essential oil was in all cases between 61.30% and 80.53%, with an average of 71.67% and a standard deviation of 5.56%. On the other hand, thymol, one of the five most abundant components in most cases, was present in a range of 0.98–2.33%, averaging 2.00% with an S.D. of 0.29%. By combining Table 1 and Table 2, it is possible to associate the values of the distillation parameters to the alterations in abundance of the two phenols. A reduction in distillation time seems to favor carvacrol and thymol extraction, at the expense of lighter compounds such as p-cymene, β-phellandrene, α-terpinene, β-myrcene and β-pinene. For distillation times of 10 min, carvacrol reaches an average of 74.24%, while for a duration of 240 min, the average abundance drops to 67.21%. A similar pattern is also observed in the case of thymol, where the average reaches 2.07% for low and intermediate times, and 1.83% for prolonged distillations. Still, this was not enough to fulfill the optimization criteria, as it turned out that a flash distillation has a counterbalancing effect on the quantity of the acquired essential oil. Thus, to achieve the objective of the present work, the three composite responses Y_1_, Y_2_ and Y_3_ were taken into account.

Carvacrol’s abundance in the essential oil was followed by the notable precursors p-cymene and γ-terpinene. The monoterpene γ-terpinene was found in the headspace area of the essential oil at a quite wide range: 5.25–13.42%, with an average of 8.91% and a standard deviation of 1.94%. Even broader was the range of the concentrations for p-cymene. In this case, the minimum relative abundance was 3.75%, with the maximum reaching 12.04% of the total aromatic profile of the essential oil, with an average at 6.91% and an S.D. of 2.38%. These two compounds are of great interest, as thymol and carvacrol are biosynthesized from γ-terpinene after a series of oxidations via p-cymene [28].

The other two monoterpenes averaging more than 0.5% of the aromatic profile were β-myrcene and α-terpinene. The first ranged between 1.62% and 4.62%, with an average of 2.69%, while the relative abundance of α-terpinene was between 0.94% and 2.54%, with an average of 1.67%. The final constituents exceeding the aforementioned threshold were the sesquiterpenes β-caryophyllene (1.02–2.65%) and β-bisabolene (0.71–2.63%).

Depending on the distillation conditions, the above eight compounds were responsible for 95.59–98.06% of the total area of the TIC chromatogram. A very small percentage (0.19–0.49%) remained unidentified, while the remaining area corresponded cumulatively to the other 13 components, i.e., β-pinene, limonene, β-phellandrene, 1-octen-3-ol, 4-thujanol, linalool, terpinen-4-ol, dihydrocarvone, α-caryophyllene, α-terpineol, endo-borneol, carvone and δ-cadinene.

### 2.3. Experimental Design Results

The experimental data were statistically analyzed, independently for each response, using analysis of variance (ANOVA), and the results are presented in Table 3. Starting with the yield (Y_1_), it can be concluded that the distillation process time (A) and the steam pressure (B) had a highly significant effect on the response, since their *p*-values were < 0.0001. Furthermore, steam temperature (C) and the quadratic term A^2^ had also a statistically significant effect on yield, since their *p*-values were < 0.05. Moving on to thymol content, Table 3 shows that distillation process time (A) and steam pressure (B) were highly significant factors (*p*-values < 0.0001), while the interactions between time with temperature (AC) and pressure with temperature (BC) were also statistically significant (*p*-values < 0.05). Finally, regarding the carvacrol content, results indicate that time (A) and pressure (B) were highly significant factors, while the interactions between time and pressure (AB), as well as between time and temperature (AC), were found to be statistically significant.

Regression models (as shown in Equations (1)–(3)) were obtained by conducting multilinear regression (MLR) model fitting. These models can be used to predict the values of the responses based on any given independent variable (steam distillation process parameter). Regarding yield, the quadratic model had the best fit, while for the other two responses a 2FI model was used. The selection of the regression model was performed aiming to fulfil all the above criteria: (i) maximum Adjusted R^2^; (ii) maximum Predicted R^2^; (iii) lowest PRESS values; (iv) adequate precision within the desirable limits (signal to noise ratio > 4); and (v) coefficient of variation with the desired value of CV < 10%.

Y_1_ = −0.396285 + (0.00586808 × A) + (0.779601 × B) + (0.00481731 × C) + (0.000621118 × A × B) + (−9.8242 × 10^−21^ × A × C) + (−0.0015873 × B × C) + (6.71403 × 10^−6^ × A^2^) + (−0.0914849 × B^2^) + (−2.21371 × 10^−5^ × C^2^) (1)

Y_2_ = −0.893557 + (0.0104069 × A) + (1.87235 × B) + (0.0211208 × C) + (0.00026938 × A × B) + (8.45723 × 10^−5^ × A × C) + (−0.0238306 × B × C) (2)

Y_3_ = −5.84111 + (0.356714 × A) + (36.5007 × B) + (0.0897288 × C) + (0.0978086 × A × B) + (0.00152101 × A × C) + (−0.128871 × B × C) (3)

**Figure 2:**
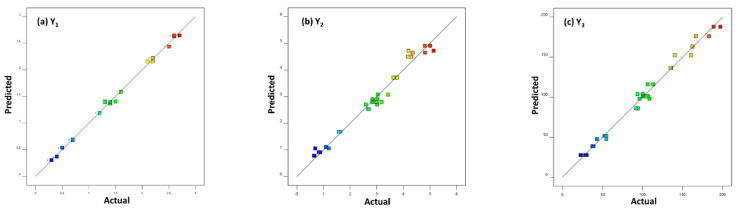
Correlation between predicted and experimental values of responses: (**a**) Y_1_ (total yield); (**b**) Y_2_ (thymol yield); (**c**) Y_3_ (carvacrol yield).

The most important criterion to evaluate any regression model is the correlation coefficient (R^2^). Based on the literature, in order for a model to be satisfactory, the R^2^ needs to be greater than 0.75 [29]. Concerning the yield (Y_1_), the quadratic model showed the highest correlation coefficient reaching up to 0.9969. On the other hand, the other two responses, thymol (Y_2_) and carvacrol (Y_3_) yield, showed similarly high R^2^ of 0.9764 and 0.9874, respectively. The excellent correlation between the model’s predicted values and the actual experimental data is also shown graphically in Figure 2.

**Figure 3:**
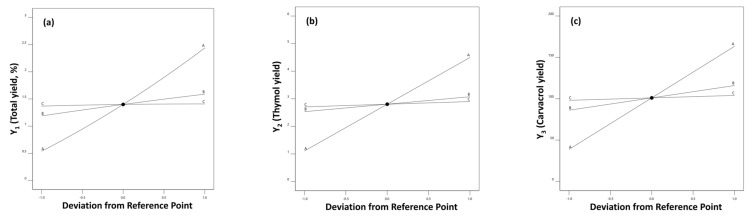
FCC perturbation plots for: (**a**) Y_1_ (total yield); (**b**) Y_2_ (thymol yield); (**c**) Y_3_ (carvacrol yield) responses. Factor A corresponds to time (min), factor B corresponds to pressure (bar) and factor C corresponds to temperature (°C).

A graphical way to illustrate and evaluate the effect of each independent factor on a specific response is to use perturbation plots. In this kind of plot, a steep slope (or curvature) corresponds to sensitivity to a specific factor. Figure 3a clearly highlights distillation process time (A) as the most critical factor affecting synergistically the distillation yield, while steam pressure (B) and temperature (C) show a slight synergistic effect. Furthermore, from Figure 3b,c, it can be concluded that thymol and carvacrol follow the same pattern with time having the most important synergistic effect, and the other two distillation parameters (pressure and temperature) having a lower synergistic effect.

**Figure 4:**
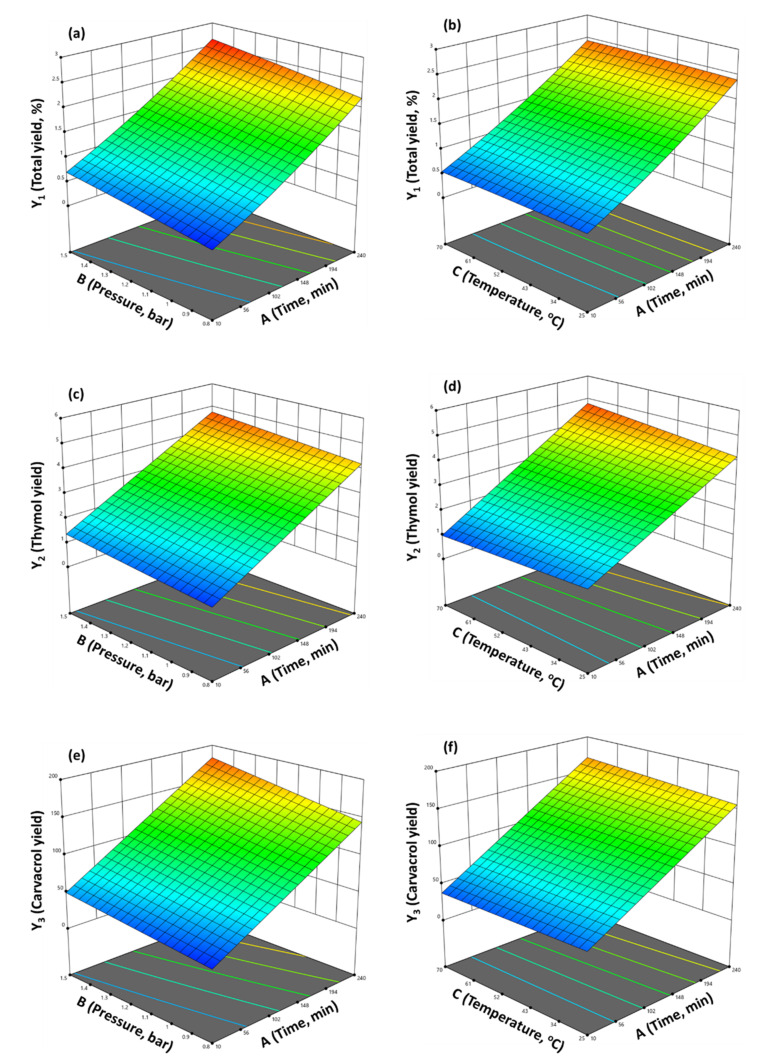
Response surfaces and contour plots for the employed CCD: (**a**) Y_1_ response to AB interaction; (**b**) Y_1_ response to AC interaction; (**c**) Y_2_ response to AB interaction; (**d**) Y_2_ response to AC interaction; (**e**) Y_3_ response to AB interaction; (**f**) Y_3_ response to AC interaction.

The effects of the main factors (A, B, and C) along with their interaction with the three responses (Y_1_, Y_2_, and Y_3_) are depicted in Figure 4 by the implementation of three-dimensional response surface plots.

Close observation of Figure 4 leads to the conclusion that the factors most affecting total yield are time and pressure. More precisely, both time and pressure have a positive correlation with total yield. On the other hand, temperature does not seem to have any significant effect on total yield. From Figure 4c,d, it can be concluded that thymol yield is also favored by high pressure and distillation duration. Temperature has a very slight effect on thymol yield. Finally, carvacrol yield is also improved when distillation is performed at high pressure and for longer. Temperature does not seem to affect carvacrol’s yield.

From the above observations, in order to achieve the highest yields (Y_1_, Y_2_, and Y_3_), one should set time and pressure at high levels, and probably also adjust temperature as low as possible to save money at industrial scale.

### 2.4. Distillation Parameters’ Optimization and Validation

**Figure 5:**
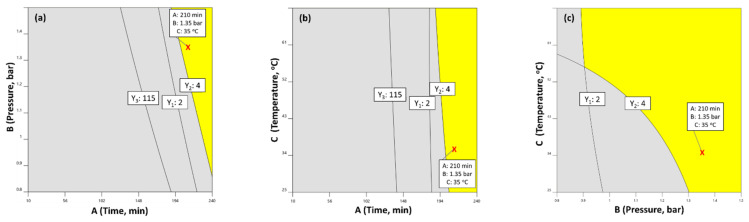
Overlay contour plot depicting the optimum DSp (yellow area). The red x-mark shows the selected optimum conditions: (**a**) distillation time 210 min, (**b**) pressure 1.35 bar, and (**c**) temperature 35 °C.

The aim of this study was to optimize the distillation conditions in order to maximize the total yield of the acquired essential oil, along with the quantity of the antioxidant components thymol and carvacrol. To achieve this, we defined the following optimization criteria: (a) the total yield of the essential oil at a pilot scale to be as high as possible (response Y_1_), (b) the yield of thymol per mass of plant material to be as high as possible (response Y_2_) and (c) the carvacrol yield per mass of plant material to be as high as possible (response Y_3_). Taking these constraints into account, we set the following goals in Design Expert^®^ software (v. 12 free trial, Stat-Ease Inc. Minneapolis, MN) to perform the graphical optimization: (i) total yield, Y_1_ ≥ 2; (ii) thymol yield, Y_2_ ≥ 4; (iii) carvacrol yield, Y_3_ ≥ 115. The bright yellow space of the overlying plot of Figure 5 illustrates the area where all the assessed criteria are satisfied.

To validate the proposed model in terms of its effectiveness to predict the responses’ values, a set of distillation parameters were chosen within the Desired Space (DSp). The conditions were chosen to maximize the yields, while keeping temperature as low as possible, with time and pressure at moderate levels within the DSp (for economic, technical and environmental reasons). The selected optimum conditions (Figure 5) were as follows: (a) distillation time 210 min, (b) pressure 1.35 bar, and (c) temperature 35 °C. Under these conditions, the model’s predictions were: 2.26% (Y_1_), 4.13 (Y_2_) and 154.30 (Y_3_). To evaluate the accuracy of the optimization procedure, a validation experiment was performed in triplicate under the aforementioned conditions, using the same plant material as previously. The validation experiment resulted in essential oil with a mean of 2.35% total yield, 4.02 thymol and 159.7 carvacrol. Experimental results were in very good agreement with the predicted values, reaching confirmation factors higher than 96% for all responses, thus verifying the effectiveness of response surface methodology to optimize the distillation process parameters for *Origanum vulgare*.

### 2.5. Antioxidant Activity

The 32 essential oil samples were evaluated for their antioxidant activities as free radical scavenging agents using the 1,1-diphenyl-2-picrylhydrazyl method and representative samples (most potent reducing agents in the DPPH assay) were tested as inhibitors of lipid peroxidation. Thymol and carvacrol are the major constituents in most of the essential oils of the Lamiaceae family [30]. However, oregano EO contains several other bioactive compounds that contribute to its antioxidant behavior, such as β-pinene, β-myrcene, α-terpinene, limonene, β-phellandrene, γ-terpinene, p-cymene, 1-octen-3-ol, 4-thujanol, linalool, α- and β-caryophyllene, terpineol, dihydrocarvone, a-terpineol, endo-borneol, β-bisabolene, carvone and δ-cadinene [31].

The formation of ROS is an unavoidable event for aerobic organisms, as a consequence of cell metabolism [32]. Due to the extreme reactivity and tendency of ROS to initiate and participate in chain reactions, the role of antioxidants as a defense system is highly recognized [33]. Antioxidants are defined as substances that, even at low concentration, significantly delay or prevent oxidation of easily oxidizable substrates.

In this assay, we measured the DPPH initial absorbance and the absorbance once the potential antioxidant had been added. The reduction of absorbance is a measure of the free DPPH due to the action of the antioxidant. The antioxidant activity was expressed as the RA% (Reducing Activity). The RA (%) values for the essential oils were low (Table 4) in comparison to the reference phenolic drug nordihydroguaiaretic acid (NDGA). However, they seemed to be time dependent and were increased by time. No significant differences in the antioxidant ability were observed in relation to the percentage of carvacrol and thymol.

Higher lipid peroxidation inhibition was shown by sample 30 and was correlated to a high percentage of carvacrol. Perusal of the results shown in Table 4 led us to present the RA% activities in three different subgroups in relation to the % content of carvacrol. In Table 5, % reducing abilities range from 37.2 to 49.5 at 20 min, whereas the % content of carvacrol assigned was from 63 to 75.4. Carvacrol % values were related to the antioxidant activity. No significant differences in the antioxidant abilities were observed in relation to the % carvacrol/thymol composition.

In Table 6, the reducing abilities RA% range from 50.4 to 51.7. These values were very similar although the % carvacrol content ranged from 68.3 to 77.5.

In Table 7, higher antioxidant activities are presented. The % carvacrol content was lower compared to the samples presented in Table 6. However, the differences in the interaction % among the samples with the free radicals were very limited.

Perusal of the antioxidant results within the three subgroups point to the role of the distillation conditions in the % values of carvacrol and thymol, especially low time (10 min), low pressure (0.80 bar) and higher temperature (70 °C).

The use of the free radical reactions’ initiator AAPH is recommended as more appropriate for measuring radical-scavenging activity in vitro, because the activity of the peroxyl radicals produced by the action of AAPH shows a greater similarity to cellular activities such as lipid peroxidation [34]. In the AAPH assay, the highly reactive alkylperoxyl radicals are intercepted mainly by a hydrogen atom transfer (HAT) from the antioxidant.

Samples 30/3, 24/6, 27/10 and 32/31 from the subgroups of Table 6 and Table 7, which presented promising interactions with DPPH, were chosen to be tested as anti-lipid peroxidation agents. Higher mean carvacrol % value was related to higher inhibition of lipid peroxidation (samples 30/3), for which the distillation conditions were less time (10 min), less pressure (0.80 bar) and low temperature.

In addition to the presence of carvacrol and thymol, which are counted as the main constituents of the EO, we must consider the fact that during the distillation some other bioactive compounds are being extracted, which also are known antioxidants, and their % values vary according to the distillation conditions. Thus, there could be a synergistic effect in terms of antioxidant activity, and this could explain the small differences in the interaction values with DPPH.

The essential oil of oregano has an important bioactive potential in its antioxidant activity and could possibly be used in the fields of natural medicines, natural food preservation, cosmetics and sanitation.

## 3. Materials and Methods

### 3.1. Materials and Instruments

In this study, dry material of *Origanum vulgare* subsp. *hirtum* plants harvested from a field in Chalkidiki, Greece (coordinates: 40°22′17.9″ N 23°15′33.1″ E), was kindly donated by Vessel Essential Oils (Thessaloniki, Greece). All pilot scale distillations were conducted at the industrial distillery “Vessel Essential Oils” and the acquired essential oils were stored in amber vials at 4 °C. The LC-MS grade methanol for sample preparation was purchased from Sigma-Aldrich (Darmstadt, Germany). The GC-MS reference standards α-terpineol, linalool, sabinene and thymol were purchased from CPAchem (Stara Zagora, Bulgaria); the 1,8-cineole, α-pinene, α-terpineol, γ-terpinene, p-cymene, linalyl acetate, terpinen-4-ol and carvacrol were purchased from Sigma-Aldrich (Darmstadt, Germany), and all were of analytical standard grade. All chemicals, solvents and biochemical reagents for the in vitro tests were of analytical grade and were purchased from commercial sources (Merck KGaA, Darmstadt, Germany; Fluka Sigma-Aldrich Laborchemikalien GmbH, Hannover, Germany). The 2,2′-Azobis(2-amidinopropane) dihydrochloride (AAPH) and sodium linoleate were obtained from Sigma Chemical, Co. (St. Louis, MO, USA).

The HS-GC/MS analysis was performed using an EVOQ GC-TQ Bruker triple quadrupole system (Bruker Daltonics, Bremen, Germany) with a CTC-PAL autosampler (CTC Analytics AG, Zwingen, Switzerland). The chromatographic separation was carried out on an HP-INNOWAX (30 m × 0.25 mm × 0.25 μm) column (Agilent Technologies, Santa Clara, CA, USA). For the in vitro tests, UV–Vis spectra were obtained on a Shimadzu Pharmaspec 1700 double-beam spectrophotometer (Shimadzu Corporation, Kyoto, Japan).

### 3.2. Experimental Design

A Face-Centered Composite design of three factors (each factor examined at three levels) was employed to investigate and optimize the main process variables that affect the steam distillate (namely, duration of the distillation, A; steam pressure, B; and steam temperature, C), as well as their interactions [35,36]. The design matrix of the employed FCC is shown in Table 1. The ranges of the independent variables were chosen based on preliminary experiments, background knowledge and practical limitations. All experiments were carried out in a randomized run order to minimize the effects of variability in the observed responses.

The evaluation of the distillation processes was achieved by optimizing the following criteria (responses): (a) the total yield of the essential oil (response Y_1_), (b) the yield of thymol per mass of plant material (response Y_2_) and (c) the carvacrol yield per mass of plant material (response Y_3_).

Multivariate data analysis using multi-linear regression (MLR) was employed and two-factor interactions (2FI) or quadratic polynomial models were fitted to the experimental data. The significance of the models was evaluated by analysis of variance (ANOVA). The quality of the fit of the polynomial model was expressed by the value of the correlation coefficient (R^2^). The main indicators demonstrating the significance and adequacy of the used model included the adequate precision (signal to noise ratio > 4), the reproducibility of the model (coefficient of variation, CV < 10%) and the predicted residual sum of square (PRESS) (values as small as possible were selected as the fittest). The optimal region of the independent variables was determined by conducting a three-dimensional response surface analysis of the independent and dependent variables [35,36].

### 3.3. Isolation of Essential Oils

The *Origanum vulgare* subsp. *hirtum* plants were collected from the whole field and they were subsequently air-dried at room temperature (18–20 °C) and under darkness. For the purposes of this study, a grade 316 stainless steel pilot scale unit was utilized. A total of 6 kg of dried plant material was used for every steam distillation process. In total, 35 distillations were carried out (32 initial distillations plus 3 verification experiments), with distillation process parameter values (i.e., distillation time, steam pressure and steam temperature) being in accordance with the implemented FCC experimental design (Table 1). The isolated essential oils were stored in amber vials at 4 °C until their analysis.

### 3.4. Headspace Gas Chromatography—Mass Spectrometry Analysis

Static headspace gas chromatography–mass spectrometry (HS-GC/MS) analysis of oregano’s essential oils was performed using an EVOQ GC-TQ Bruker triple quadrupole system (Bruker Daltonics) with a CTC-PAL autosampler (CTC Analytics AG). The chromatographic separation was carried out on an HP-INNOWAX (30 m × 0.25 mm × 0.25 μm) column (Agilent Technologies).

To obtain sufficient peak resolution, satisfactory peak shapes and increased intensity (avoiding column overload), a series of preliminary experiments were carried out, testing different analytical parameters. For this purpose, a pooled QC sample was prepared by mixing equal volumes (50 μL) from each sample. Initially, a sample’s dilution ratio and final volume were selected by testing different dilution ratios of essential oil in methanol—1:1000, 1:500 and 1:20 (*v*/*v*)—and volumes of either 20 μL or 50 μL. Next, various thermal gradients were applied for the analysis of the QC sample, considering the literature and previous knowledge [37]. Finally, MS conditions, along with the main factors that affect HS injection (i.e., incubation temperature, incubation time, agitator speed, injection volume and injection flow rate) were considered and optimized.

The 32 essential oils acquired from the experimental design were eventually diluted in methanol at a ratio of 1:500 (*v*/*v*). After vortexing, 20 μL was pipetted in a 20 mL autosampler headspace vial to be analyzed by HS-GC/MS. Reference standards were also diluted in methanol and then mixed together into a final concentration of 25 ppm per substance, 20 μL of which was also pipetted in a 20 mL headspace vial for GC-MS analysis.

Helium (99.999%), as a carrier gas, was set at a constant flow rate of 1 mL/min. A split injection mode was applied at a ratio of 1:10 for the first 0.01 min and 1:100 after 1 min. Before injection of the sample, incubation was performed at 90 °C for 15 min, with the agitator speed at 500 rpm. Injection volume was set at 1000 μL and flow rate at 2 mL/min. Inlet temperature was set at 250 °C.

A thermal gradient was selected to provide adequate peak separation. The initial oven temperature was set at 52 °C, where it remained for 2 min, then increased with a 5 °C/min rate to 80 °C, held at 80 °C for 4 min, and finally increased with a 4 °C/min rate to 250 °C, where it remained for 1 min. The MS transfer line temperature was set at 250 °C, while the ion source temperature was set at 230 °C. Fragmentation was performed by applying electron impact (EI) ionization at 70 eV. Full scan spectra were acquired from 25 to 500 amu, with a 250 ms scan time and a collection delay of 3.8 min.

### 3.5. Data Processing and Analysis

Chromatographic data were treated using MSWS 8 data process software (Bruker Daltonics, Billerica, MA, USA) and identification was performed using the NIST17 Mass Spectral Library (mainlib and replib EI Databases), in synergy with the existing literature and reference standards’ retention times and mass spectra. Deconvolution of complex peaks was performed by utilizing the AMDIS program (Automated Mass Spectral Deconvolution and Identification System, NIST), while peak integration of the total ion chromatogram was performed manually.

For experimental design, RStudio (v.1.3.959, RStudio, PBC, Boston, MA, USA) in combination with Design Expert^®^ (v.12 free trial, Stat-Ease Inc., Minneapolis, MN, USA) was used for graphical optimization [36,38].

The experiments were carried out in a randomized order. For the verification experiment, the results of the three independent experiments are given as mean value ± standard deviation (S.D.). Statistical significance in the differences of the means was evaluated by using Student’s *t*-test or one-way ANOVA (Tukey and Scheffe tests) for the single and multiple comparisons of experimental groups, respectively. A difference with *p*-value < 0.05 was considered statistically significant. All statistical analyses were performed using SPSS Statistics (v.25.0, IBM, Armonk, NY, USA).

### 3.6. Biological In Vitro Assays

For the in vitro assays, a stock solution at a concentration of 20 µL in 200 µL of absolute ethanol was used, from which several dilutions were made. The measurements were performed at least in triplicate and the standard deviation of absorbance was less than 10% of the mean. Statistical comparisons were made using the Student’s *t*-test. A statistically significant difference was defined as *p* < 0.05.

### 3.7. Interaction with the Stable Radical 1,1-Diphenyl-picrylhydrazyl (DPPH) 

The 2,2-diphenyl-1-picrylhydrazyl is a quick and easy assay for the measurement of antioxidant properties. The test is associated with the elimination of DPPH, which would be a stabilized free radical. The free radical DPPH interacts with an odd electron to yield a strong absorbance at 517 nm (purple). An antioxidant reacts to DPPH to form DPPHH, which has a lower absorbance than DPPH, because of the lower amount of hydrogen. The solution decolorizes, as the number of electrons absorbed increases. As soon as the DPPH solutions are combined with the hydrogen atom source, the lower state of diphenylpicrylhydrazine is formed, shedding its violet color [39,40,41].

To a solution of DPPH 1 mL (50 μΜ) in absolute ethanol, the appropriate volume of the essential oils (20 μL from the stock solution containing 20 µL/200 µL) dissolved in absolute ethanol was added. The mixture was shaken vigorously and in some cases with the help of ultrasound and allowed to stand for 20 min or 60 min; absorbance at 517 nm was determined spectrophotometrically and the percentage of activity was calculated. For the calculation of the in vitro antioxidant assays, the formula **(A_0_ − A_1_)/A_0_ × 100**, was used, where A_0_ is the control absorbance and A_1_ is the sample absorbance [39,40,41].

### 3.8. Inhibition of Linoleic Acid Peroxidation 

An in vitro study was performed as previously reported. Production of conjugated diene hydroperoxide by oxidation of linoleic acid in an aqueous dispersion is monitored at 234 nm. AAPH is used as a free radical initiator. This assay can be used to follow oxidative changes and to understand the contribution of each tested compound. Azo compounds generating free radicals through spontaneous thermal decomposition are useful for in vitro studies of free radical production. The water-soluble azo compound AAPH has been extensively used as a clean and controllable source of thermally produced alkylperoxyl free radicals. The tested essential oils as stock solutions were dissolved in absolute ethanol (20 µL/200 µL). Ten microliters of the 16 mM sodium linoleate solution were added to the UV cuvette containing 0.93 mL of 0.05 M phosphate buffer (pH 7.4), prethermostatted at 37 °C. The oxidation reaction was initiated at 37 °C under air by the addition of 50 µL of 40 mM AAPH solution, which is used as a free radical initiator. Oxidation was carried out in the presence of samples (10 µL) in the assay without antioxidant and monitored at 234 nm. Lipid oxidation was recorded in the presence of the same level of ethanol. Trolox was used as the appropriate standard. Lipid peroxidation inhibition was expressed as inhibition percentage and was calculated using the formula **(A_0_ − A_1_)/A_0_ × 100**, where A_0_ is the control absorbance and A_1_ is the sample absorbance [39,40,41].

## 4. Conclusions

In the present work, the three adjustable parameters of steam distillation at an industrial scale (distillation time, steam pressure and temperature) were optimized by a Face-Centered Composite experimental design, in order to obtain essential oils of high added value from the plant *Origanum vulgare* subsp. *hirtum* (Greek oregano). The evaluation of optimum values was performed by considering the following responses: the plant’s yield in essential oil, content of carvacrol and content of thymol (as determined by means of headspace GC-MS).

The lowest yield obtained was 0.3% *w*/*w*, while the highest one reached the value of 2.7% *w*/*w*. The 32 independent distillation processes averaged a yield of 1.44%, with a standard deviation of 0.78%, displaying perfect reproducibility among samples distilled under identical conditions. The relative abundance of carvacrol in the present work was between 61.30% and 80.53%, with an average of 71.67% and a standard deviation of 5.56%. Thymol was found in a range of 0.98–2.33%, averaging 2.00% with a standard deviation of 0.29%.

The three responses seem to follow the same pattern: distillation time (A) crucially affects each one of the responses in a synergistic way, while steam pressure (B) and temperature (C) exhibit only a slight synergistic effect. It was concluded that the factor that most affects the three responses is time, followed by pressure. Both factors display a positive correlation with the three responses. On the other hand, temperature has no clear impact on any of the three responses. Therefore, to achieve the highest possible yields, distillation time and steam pressure should be set at high levels, regulating temperature as low as possible at the same time, for financial and environmental reasons. Taking all the above into account, a graphical optimization was performed by setting the following goals: (i) total yield, Y_1_ ≥ 2; (ii) thymol yield, Y_2_ ≥ 4; (iii) carvacrol yield, Y_3_ ≥ 115.

Finally, to validate the proposed model in terms of its effectiveness to predict the responses’ values, a set of distillation parameters were chosen within the Desired Space. The conditions were chosen to maximize the three responses, keeping temperature as low as possible, while selecting moderate values within the Desired Space for the other two factors (time and pressure). The conditions’ values were selected as follows: (a) distillation time 210 min, (b) steam pressure 1.35 bar, and (c) temperature 35 °C. Under these conditions, the model’s predictions were: 2.26% (Y_1_), 4.13 (Y_2_) and 154.30 (Y_3_). The validation experiment resulted in essential oil with a mean of 2.35% total yield, and a score of 4.02 for thymol and 159.7 for carvacrol. These experimental results were in very good agreement with the predicted values, reaching confirmation factors higher than 96% for all responses, verifying the effectiveness of the response surface methodology in optimizing the distillation process parameters for Greek oregano.

As for the in vitro antioxidant activity of the essential oils with the DPPH method, no clear correlation could be established between the antioxidant activities and the respective contents in thymol and carvacrol. However, one should consider the fact that, except for the major participation of carvacrol and thymol, these essential oils contain some additional bioactive constituents, with their relative abundance varying in accordance with the distillation conditions. Thus, in terms of antioxidant activity, a synergistic effect should also be attributed, potentially explaining minor differences in the interaction values with DPPH.

Finally, the essential oils displaying the most promising interaction with DPPH were also tested as inhibitors of lipid peroxidation. The highest lipid peroxidation inhibition was shown by samples corresponding to a high percentage of carvacrol. Distillation conditions for these samples were low time (10 min), minimum pressure (0.80 bar) and very low temperature (25 °C).

Evaluating the impact of the distillation conditions on the in vitro results, it seems that lower pressure, less time and maybe higher temperature are crucial for enhanced antioxidant activities. Due to their considerable antioxidant potential, these oils could possibly be used in the fields of natural medicine, natural food preservation, cosmetics and sanitation.

## Figures and Tables

**Table 1:** Matrix and observed responses of the applied FCC experimental design.

	Factors			Responses		
Sample	A Time (min)	B Pressure (bar)	C Temperature (°C)	Y_1_ Total Yield (% *w*/*w*)	Y_2_ Thymol Yield (‱)	Y_3_ Carvacrol Yield (‱)
30	10	0.80	25.0	0.3	0.66	22.99
3	10	0.80	25.0	0.3	0.64	22.50
19	240	0.80	25.0	2.1	3.76	135.55
11	240	0.80	25.0	2.2	3.63	134.85
26	10	1.50	25.0	0.7	1.63	52.34
20	10	1.50	25.0	0.7	1.57	54.81
24	240	1.50	25.0	2.6	4.81	182.76
6	240	1.50	25.0	2.6	4.35	166.40
27	10	0.80	70.0	0.4	0.88	30.28
10	10	0.80	70.0	0.4	0.82	28.24
9	240	0.80	70.0	2.2	5.13	140.03
17	240	0.80	70.0	2.2	4.19	160.49
32	10	1.50	70.0	0.7	0.69	54.75
31	10	1.50	70.0	0.7	1.20	42.91
12	240	1.50	70.0	2.7	4.81	188.59
22	240	1.50	70.0	2.6	5.00	196.97
4	10	1.15	47.5	0.5	1.10	37.29
18	10	1.15	47.5	0.5	1.08	38.67
1	240	1.15	47.5	2.5	4.30	162.26
23	240	1.15	47.5	2.5	4.18	162.17
16	125	0.80	47.5	1.2	2.68	94.19
5	125	0.80	47.5	1.2	2.70	91.79
13	125	1.50	47.5	1.6	3.42	113.26
2	125	1.50	47.5	1.6	3.04	106.37
15	125	1.15	25.0	1.4	3.01	108.46
28	125	1.15	25.0	1.4	2.59	96.26
25	125	1.15	70.0	1.3	2.84	93.65
21	125	1.15	70.0	1.5	3.02	100.24
8	125	1.15	47.5	1.4	2.84	100.72
7	125	1.15	47.5	1.4	3.18	106.45
29	125	1.15	47.5	1.4	2.96	102.28
14	125	1.15	47.5	1.3	2.99	104.69

**Table 2:** Detected compounds by headspace GC-MS and their relative abundance * in the volatile fraction of the 32 oregano essential oils.

Compound	RT (min)	RI Polar (NIST) **	Sample															
			30	3	19	11	26	20	24	6	27	10	9	17	32	31	12	22
β-Pinene	5.09	1112 ± 7	0.00%	0.00%	0.17%	0.19%	0.00%	0.00%	0.13%	0.16%	0.00%	0.00%	0.22%	0.17%	0.09%	0.15%	0.24%	0.18%
β-Myrcene	6.36	1161 ± 7	1.65%	1.78%	3.61%	4.15%	2.08%	1.62%	3.13%	4.16%	1.70%	2.14%	3.93%	2.77%	2.11%	3.43%	3.39%	2.66%
α-Terpinene	6.71	1180 ± 8	1.41%	1.45%	2.19%	2.54%	1.55%	1.12%	1.90%	2.37%	1.37%	1.84%	2.40%	1.68%	1.49%	2.45%	1.97%	1.53%
Limonene	7.19	1200 ± 7	0.14%	0.15%	0.22%	0.32%	0.15%	0.14%	0.16%	0.14%	0.12%	0.17%	0.32%	0.20%	0.20%	0.34%	0.20%	0.15%
β-Phellandrene	7.45	1211 ± 7	0.12%	0.14%	0.25%	0.39%	0.16%	0.09%	0.26%	0.36%	0.11%	0.16%	0.37%	0.20%	0.20%	0.34%	0.23%	0.19%
γ-Terpinene	8.61	1246 ± 9	8.19%	9.24%	10.61%	11.43%	7.72%	7.01%	8.95%	11.00%	9.07%	11.68%	11.50%	8.24%	7.48%	13.42%	8.77%	6.68%
p-Cymene	9.53	1272 ± 8	3.87%	3.86%	8.48%	12.04%	4.69%	3.82%	8.28%	10.57%	3.75%	4.74%	9.71%	6.92%	5.76%	8.16%	8.93%	6.73%
1-Octen-3-ol	16.89	1450 ± 7	0.21%	0.31%	0.49%	0.55%	0.32%	0.30%	0.47%	0.57%	0.30%	0.35%	0.54%	0.42%	0.23%	0.55%	0.41%	0.33%
4-Thujanol	17.27	1465 ± 9	0.10%	0.14%	0.37%	0.36%	0.20%	0.15%	0.39%	0.48%	0.13%	0.14%	0.41%	0.32%	0.12%	0.27%	0.36%	0.30%
Linalool	20.40	1547 ± 7	0.07%	0.10%	0.13%	0.09%	0.10%	0.08%	0.10%	0.12%	0.09%	0.09%	0.11%	0.10%	0.05%	0.13%	0.09%	0.08%
β-Caryophyllene	21.77	1595 ± 16	1.67%	1.76%	2.65%	1.85%	2.00%	1.54%	1.51%	1.73%	1.69%	1.95%	1.74%	1.47%	1.14%	2.49%	1.47%	1.34%
Terpinen-4-ol	22.05	1602 ± 9	0.52%	0.55%	0.76%	0.71%	0.61%	0.49%	0.56%	0.64%	0.53%	0.62%	0.61%	0.51%	0.33%	0.82%	0.52%	0.46%
Dihydrocarvone	22.16	1624 ± 21	0.07%	0.09%	0.11%	0.09%	0.08%	0.08%	0.07%	0.08%	0.09%	0.09%	0.07%	0.07%	0.06%	0.15%	0.07%	0.07%
α-Caryophyllene	24.08	1667 ± 14	0.18%	0.17%	0.23%	0.13%	0.21%	0.13%	0.12%	0.12%	0.17%	0.16%	0.12%	0.11%	0.08%	0.19%	0.09%	0.10%
α-Terpineol	25.05	1697 ± 10	0.12%	0.11%	0.24%	0.13%	0.18%	0.11%	0.15%	0.13%	0.12%	0.13%	0.14%	0.14%	0.06%	0.26%	0.15%	0.16%
endo-Borneol	25.15	1702 ± 15	0.25%	0.29%	0.45%	0.35%	0.32%	0.27%	0.34%	0.39%	0.27%	0.33%	0.37%	0.31%	0.15%	0.40%	0.34%	0.31%
β-Bisabolene	25.97	1727 ± 11	2.02%	2.07%	2.05%	1.21%	1.96%	1.97%	0.94%	0.87%	2.03%	2.10%	1.01%	1.12%	0.87%	2.63%	0.77%	0.71%
Carvone	26.06	1740 ± 12	0.04%	0.06%	0.10%	0.04%	0.06%	0.07%	0.08%	0.08%	0.05%	0.05%	0.05%	0.06%	0.02%	0.10%	0.08%	0.08%
δ-Cadinene	26.83	1758 ± 13	0.20%	0.20%	0.16%	0.09%	0.19%	0.18%	0.05%	0.05%	0.18%	0.18%	0.07%	0.08%	0.08%	0.22%	0.04%	0.04%
Thymol	38.04	2189 ± 9	2.20%	2.14%	1.79%	1.65%	2.33%	2.24%	1.85%	1.67%	2.19%	2.05%	2.33%	1.91%	0.98%	1.71%	1.78%	1.92%
Carvacrol	38.69	2236 ± 10	76.63%	74.98%	64.55%	61.30%	74.77%	78.30%	70.29%	64.00%	75.69%	70.60%	63.65%	72.95%	78.22%	61.30%	69.85%	75.76%
**Compound**	**RT (min)**	RI Polar (NIST) **	**Sample**															
			**4**	**18**	**1**	**23**	**16**	**5**	**13**	**2**	**15**	**28**	**25**	**21**	**8**	**7**	**29**	**14**
β-Pinene	5.09	1112 ± 7	0.00%	0.00%	0.22%	0.23%	0.08%	0.09%	0.21%	0.19%	0.15%	0.18%	0.17%	0.24%	0.20%	0.17%	0.16%	0.13%
β-Myrcene	6.36	1161 ± 7	1.88%	1.81%	3.88%	3.77%	1.66%	1.97%	2.91%	3.34%	2.23%	3.23%	2.56%	3.39%	2.83%	2.16%	2.46%	1.67%
α-Terpinene	6.71	1180 ± 8	1.31%	1.22%	2.02%	1.96%	0.99%	1.13%	1.48%	2.08%	1.32%	1.91%	1.62%	2.01%	1.57%	1.26%	1.37%	0.94%
Limonene	7.19	1200 ± 7	0.11%	0.11%	0.34%	0.26%	0.13%	0.10%	0.16%	0.24%	0.19%	0.23%	0.18%	0.21%	0.29%	0.20%	0.20%	0.16%
β-Phellandrene	7.45	1211 ± 7	0.10%	0.10%	0.32%	0.34%	0.13%	0.10%	0.19%	0.27%	0.21%	0.29%	0.20%	0.26%	0.29%	0.22%	0.23%	0.17%
γ-Terpinene	8.61	1246 ± 9	8.51%	7.40%	11.07%	11.19%	6.52%	7.37%	6.70%	10.82%	7.28%	10.03%	8.82%	10.53%	6.94%	7.32%	8.32%	5.25%
p-Cymene	9.53	1272 ± 8	4.25%	4.75%	9.99%	10.12%	4.74%	4.72%	9.79%	9.07%	5.50%	8.45%	6.73%	7.84%	8.22%	5.48%	6.43%	4.58%
1-Octen-3-ol	16.89	1450 ± 7	0.27%	0.28%	0.53%	0.50%	0.30%	0.27%	0.29%	0.44%	0.21%	0.46%	0.38%	0.36%	0.45%	0.26%	0.40%	0.23%
4-Thujanol	17.27	1465 ± 9	0.14%	0.15%	0.37%	0.44%	0.19%	0.15%	0.24%	0.33%	0.15%	0.34%	0.25%	0.22%	0.29%	0.17%	0.26%	0.17%
Linalool	20.40	1547 ± 7	0.10%	0.08%	0.12%	0.11%	0.07%	0.08%	0.09%	0.10%	0.05%	0.09%	0.09%	0.09%	0.11%	0.07%	0.10%	0.07%
β-Caryophyllene	21.77	1595 ± 16	2.13%	1.37%	1.76%	1.77%	1.39%	1.80%	1.81%	1.76%	1.02%	1.50%	1.67%	2.34%	1.75%	1.62%	1.76%	1.32%
Terpinen-4-ol	22.05	1602 ± 9	0.58%	0.49%	0.59%	0.62%	0.51%	0.50%	0.64%	0.62%	0.37%	0.54%	0.56%	0.51%	0.60%	0.42%	0.54%	0.43%
Dihydrocarvone	22.16	1624 ± 21	0.09%	0.09%	0.09%	0.09%	0.07%	0.08%	0.10%	0.10%	0.06%	0.09%	0.09%	0.10%	0.10%	0.07%	0.08%	0.06%
α-Caryophyllene	24.08	1667 ± 14	0.22%	0.13%	0.12%	0.13%	0.12%	0.17%	0.14%	0.13%	0.07%	0.09%	0.13%	0.22%	0.16%	0.13%	0.15%	0.12%
α-Terpineol	25.05	1697 ± 10	0.15%	0.15%	0.14%	0.15%	0.16%	0.15%	0.16%	0.16%	0.15%	0.14%	0.18%	0.18%	0.16%	0.15%	0.15%	0.15%
endo-Borneol	25.15	1702 ± 15	0.28%	0.27%	0.34%	0.39%	0.30%	0.26%	0.24%	0.34%	0.28%	0.37%	0.36%	0.32%	0.30%	0.21%	0.29%	0.23%
β-Bisabolene	25.97	1727 ± 11	2.48%	1.56%	1.01%	0.93%	1.42%	1.84%	1.41%	1.14%	0.83%	1.03%	1.32%	1.78%	1.26%	1.38%	1.48%	1.12%
Carvone	26.06	1740 ± 12	0.06%	0.06%	0.08%	0.07%	0.06%	0.07%	0.06%	0.06%	0.04%	0.06%	0.06%	0.08%	0.06%	0.05%	0.05%	0.04%
δ-Cadinene	26.83	1758 ± 13	0.22%	0.16%	0.07%	0.07%	0.15%	0.16%	0.11%	0.09%	0.06%	0.06%	0.10%	0.16%	0.10%	0.10%	0.12%	0.11%
Thymol	38.04	2189 ± 9	2.21%	2.17%	1.72%	1.67%	2.23%	2.25%	2.14%	1.90%	2.15%	1.85%	2.18%	2.01%	2.03%	2.27%	2.12%	2.30%
Carvacrol	38.69	2236 ± 10	74.57%	77.34%	64.91%	64.87%	78.49%	76.49%	70.79%	66.48%	77.47%	68.76%	72.04%	66.82%	71.95%	76.04%	73.05%	80.53%

* Refers to the percentage of peak’s area over the total area of the TIC. ** RI Polar (NIST): Retention indices of the respective compounds according to NIST17 Mass Spectral Library (for polar columns).

**Table 3:** ANOVA results for the employed CCD experimental design (insignificant factors were eliminated for convenience of presentation).

	Responses *					
	Y_1_		Y_2_		Y_3_	
Factors **	F-Value	*p*-Value ***	F-Value	*p*-Value ***	F-Value	*p*-Value ***
A	6762.13	<0.0001	982.38	<0.0001	1832.17	<0.0001
B	299.71	<0.0001	25.12	<0.0001	105.08	<0.0001
C	-	-	-	-	-	-
AB	-	-	-	-	5.86	0.0231
AC	-	-	13.11	0.0013	5.86	0.0231
BC	-	-	9.64	0.0047	-	-
A^2^	15.57	0.0007	-	-	-	-
R^2^	0.9969		0.9764		0.9874	

* Responses: Y_1_, total yield; Y_2_, thymol yield; Y_3_, carvacrol yield. ** Factors: A, distillation process time; B, steam pressure; C, steam temperature. *** *p*-Values less than 0.0500 indicate model terms are significant. Values greater than 0.1000 indicate the model terms are not significant.

**Table 4:** In vitro antioxidant activity, as % reducing activity values (RA%) and % inhibition of lipid peroxidation values, for the 32 essential oils in correlation with the abundance of oregano’s main phenolic monoterpenes.

Sample *	% Inhibition of Lipid Peroxidation (± SD)	% Interaction with DPPH–RA% (Reducing Activity) 20 min (± SD)	% Interaction with DPPH–RA% (Reducing Activity) 60 min (± SD)	Carvacrol (%) **	Thymol (%) **
30	80 ± 1.1	52.7 ± 1.2	77.2 ± 2.3	76.63	2.20
3		49.7 ± 0.9	64.4 ± 2.7	74.98	2.14
19		49.3 ± 0.8	64.9 ± 1.8	64.55	1.79
11		48.3 ± 1.3	63.2 ± 2.1	61.30	1.65
26		54.5 ± 0.8	66.0 ± 2.4	74.77	2.33
20		48.9 ± 075	58.2 ± 0.7	78.30	2.24
24	33 ± 0.03	61.0 ± 1.3	80.0 ± 2.9	70.29	1.85
6		58.0 ± 0.5	69.0 ± 1.1	64.00	1.67
27	3 ± 0.001	56.0 ± 1.3	70.0 ± 1.9	75.69	2.19
10		57.0 ± 1.1	68.0 ± 0.7	70.60	2.05
9		49.0 ± 0.4	62.0 ± 0.4	63.65	2.33
17		51.8 ± 03	69.0 ± 0.2	72.95	1.91
32	15 ± 0.01	61.4 ± 1.7	61.0 ± 1.0	78.22	0.98
31		62.4 ± 2.2	73.0 ± 1.7	61.30	1.71
12		58.3 ± 2.0	69.0 ± 0.6	69.85	1.78
22		58.6 ± 1.1	68.0 ± 1.6	75.76	1.92
4		53.4 ± 0.5	66.0 ± 1.3	74.57	2.21
18		48.2 ± 0.3	61.0 ± 0.4	77.34	2.17
1		47.5 ± 0.2	61.0 ± 0.7	64.91	1.72
23		50.4 ± 0.9	66.0 ± 1.6	64.87	1.67
16		56.2 ± 1,0	67.0 ± 1.4	78.49	2.23
5		45.0 ± 0.4	62.0 ± 2.1	76.49	2.25
13		39.1 ± 0.6	51.0 ± 1.1	70.79	2.14
2		45.7 ± 0.4	65.0 ± 0.9	66.48	1.90
15		45.5 ± 0.9	65.0 ± 1.0	77.47	2.15
28		47.4 ± 1.3	65.0 ± 1.4	68.76	1.85
25		30.0 ± 0.8	45.0 ± 0.7	72.04	2.18
21		47.4 ± 1.4	65.0 ± 1.1	66.82	2.01
8		49.6 ± 1.6	65.0 ± 0.7	71.95	2.03
7		53.4 ± 2.0	69.0 ± 1.6	76.04	2.27
29		46.1 ± 0.7	63.0 ± 1.2	73.05	2.12
14		49.3 ± 0.3	68.0 ± 2.4	80.53	2.30
NDGA		88.0 ± 2.3	93.0 ± 3.2		
Trolox	92 ± 1.9				

* See Table 1 for the respective factors of the 32 independent experiments. ** Relative abundance in the volatile fraction of the oregano’s essential oil.

**Table 5:** Antioxidant activity (mean values of RA% values) in correlation with the mean % values of carvacrol content and with the distillation process factors applied.

Samples	RA% 20 Min	RA% 60 Min	Mean Values of % Carvacrol	Time (Min)	Pressure (Bar)	Temperature (°C)
8/7/29/14	49.5	66.3	75.4	125	1.15	47.5
15/28	46.5	65.0	73.0	125	1.15	25.0
25/21	37.2	55.0	69.4	125	1.15	70.0
2/13	42.4	58.0	68.6	125	1.50	47.5
1/23	49.0	63.5	64.9	240	1.15	47.5
11/19	48.8	64.1	63.0	240	0.80	25.0

**Table 6:** Antioxidant activity (mean values of RA% values) in correlation with the mean % values of carvacrol content and with the distillation process factors applied.

Samples	RA% 20 Min	RA% 60 Min	Mean Values of % Carvacrol	Time (Min)	Pressure (Bar)	Temperature (°C)
16/5	51.0	64.5	77.5	125	0.80	47.5
26/20	51.7	62.0	76.6	10	1.50	25.0
4/18	51.0	63.5	76.0	10	1.15	47.5
30/3	51.2	70.8	75.8	10	0.80	25.0
9/17	50.4	65.5	68.3	240	0.80	70.0

**Table 7:** Antioxidant activity (mean values of RA% values) in correlation with the mean % values of carvacrol content and with the distillation process factors applied.

**Samples**	**RA% 20 Min**	**RA% 60 Min**	**Mean** **Values of** **% Carvacrol**	**Time (Min)**	**Pressure (** **Bar** **)**	**Temperature (°C)**
27/10	56.5	69.0	74.6	10	0.80	70.0
12/22	58.5	68.5	72.8	240	1.50	70.0
32/31	62.2	67.0	69.8	10	1.50	70.0
24/6	59.5	74.5	67.0	240	1.50	25.0

## Data Availability

Not applicable.

## Sample Availability

Samples of the essential oils are available from the authors.
